# Housing gaps, mosquitoes and public viewpoints: a mixed methods assessment of relationships between house characteristics, malaria vector biting risk and community perspectives in rural Tanzania

**DOI:** 10.1186/s12936-018-2450-y

**Published:** 2018-08-17

**Authors:** Emmanuel W. Kaindoa, Marceline Finda, Jepchirchir Kiplagat, Gustav Mkandawile, Anna Nyoni, Maureen Coetzee, Fredros O. Okumu

**Affiliations:** 1 Environmental Health and Ecological Science Department, Ifakara Health Institute, P. O. Box 53, Ifakara, Tanzania; 2 School of Public Health, Faculty of Health Sciences, University of the Witwatersrand, Johannesburg, South Africa; 3 Institute of Biodiversity, Animal Health and Comparative Medicine, University of Glasgow, Glasgow, G12 8QQ UK; 4 School of Medicine, College of Health Sciences, Moi University, Eldoret, Kenya; 5 Wits Research Institute for Malaria and Wits/MRC Collaborating Centre for Multidisciplinary Research on Malaria, School of Pathology, Faculty of Health Sciences, University of the Witwatersrand, Johannesburg, South Africa; 6 Centre for Emerging Zoonotic & Parasitic Diseases, National Institute for Communicable Diseases, Johannesburg, South Africa

**Keywords:** Community knowledge, Malaria transmission, Housing characteristics, Environmental features, Mosquitoes, Tanzania

## Abstract

**Background:**

House improvement and environmental management can significantly improve malaria transmission control in endemic communities. This study assessed the influence of physical characteristics of houses and surrounding environments on mosquito biting risk in rural Tanzanian villages, and examined knowledge and perceptions of residents on relationships between these factors and malaria transmission. The study further assessed whether people worried about these risks and how they coped.

**Methods:**

Entomological surveys of indoor mosquito densities were conducted across four villages in Ulanga district, south-eastern Tanzania. The survey involved 48 sentinel houses sampled monthly and other sets of 48 houses randomly recruited each month for one-off sampling over 12 months. Physical characteristics of the houses and surrounding environments were recorded. Questionnaire surveys were administered to 200 household heads to assess their knowledge and concerns regarding the observed housing and environmental features, and whether they considered these features when constructing houses. Focus group discussions, were conducted to clarify emergent themes on people’s perceptions on relationships between housing or environmental factors and malaria transmission.

**Results:**

The entomological surveys showed statistically higher indoor densities of the malaria vectors (*Anopheles arabiensis* and *Anopheles funestus*) in houses with mud walls compared to plastered or brick walls, open eaves compared to closed eaves and unscreened windows compared to screened windows. Most respondents reported that their houses allowed mosquito entry, at least partially. Participants were aware that house structure and environmental characteristics influenced indoor mosquito densities and consequently malaria transmission. They were concerned about living in poorly-constructed houses with gaps on eaves, walls, windows and doors but were constrained by low income.

**Conclusion:**

In rural south-eastern Tanzania, significant proportions of people still live in houses with open eaves, unscreened windows and gaps on doors. Though they are fully aware of associated mosquito biting and pathogen transmission risks, they are constrained by low-income levels. The study proposes that community-based house improvement initiatives combined with targeted subsidies could lower the financial barriers, improve access to essential construction materials or designs, and significantly accelerate malaria transmission control in these communities.

## Background

The WHO 2017 World Malaria Report showed significant successes in malaria control in various countries, but with an overall slight increase in malaria cases and deaths relative to the previous year [1]. Over the past decades, LLINs and IRS have been widely used alongside improved diagnosis and treatment, primarily with artemisinin-based combination therapy. These tools have contributed most of the gains accrued against malaria in the past decade [1, 2], but evidence suggests that new interventions will be required to finally move to zero transmission [3, 4]. Unfortunately, novel strategies, such as improved housing [5] and environmental management for malaria control, have only rarely considered [6, 7]. Tanzania has experienced more than 50% reduction in malaria prevalence in children aged 2–10 years across the country since 2000 [8]. It is estimated that less than 2% of people in mainland Tanzania now live in places considered as having intense year-round transmission, while more than 60% of Tanzanians live in areas considered as having low transmission [8]. The 2015–2016 malaria indicator surveys reported an increase in average malaria prevalence to 14.8% prevalence in children under 5 years [9], up from 9.5% in 2012 [10], but the most recent survey has shown much reduced prevalence of 7.3% [11]. Despite these gains, there are still numerous challenges, among them, the rise of insecticide resistance [12], sub-optimal net use each night [9], and the high costs. Greater vigilance and additional interventions are therefore required to consolidate past gains and accelerate efforts towards the current national malaria control targets and eventual elimination.

Community involvement in vector control programmes is essential for successful malaria transmission reduction [13–16]. Existing evidence suggests that acceptance and optimal use of interventions, such as indoor residual spraying (IRS) and long-lasting insecticide-treated nets (LLINs) depends on the level of education and awareness in target communities [17], as well as grass-root organization of such communities to support the health initiatives [16]. Similar successes have been observed with other vector control approaches such as larval source management. For example, in Tanzania, a large-scale community-based larvicide application programme in the city of Dar es Salaam, significantly reduced malaria infection prevalence among city residents [18], despite significant logistical and coverage challenges. In that programme, community owned resource persons supported specific efforts such as mapping, larvicide application and vector surveillance [19, 20]. Elsewhere, in an interesting integration of farmers’ experiences, van den Berg and Knols [21] highlighted the importance and potential of using farmer field schools as a method for improving community participation in malaria control. They suggested that malaria control can benefit from complementarities between rural development, farming and pest management and argued for a form of education that uses experiential learning methods to build farmers experiences, thereby achieving improvements in both health and development [21].

Unfortunately, despite the substantial evidence on benefits of community engagement in malaria prevention and various innovations on how to achieve this goal, community participation is increasingly relegated, as malaria-endemic countries adopt vertical campaigns such as universal LLIN distributions and mass drug administration in some settings. Instead, the knowledge and experiences of recipient communities is rarely sought even where such additional knowledge would significantly improve outcomes. In rural Ulanga district, south-eastern Tanzania, recent studies have demonstrated that community members were either at par or more knowledgeable than experts in identifying locations where mosquitoes are most abundant [22] and locations where male mosquitoes regularly swarm [23]. Both examples emphasize the potential benefits of relying on communities for improved vector control.

Another important question is whether individuals understand how malaria transmission risk varies across their communities and how it is affected by factors such as housing characteristics and the environment. Evidence suggests that transmission intensities are rarely uniform between districts and villages or even between households in the same village. Instead some households are always disproportionally more exposed to biting risk and malaria infections than community averages [24]. In a recent study, it was found that there is strong correlations between household occupancy and indoor densities of common malaria vectors (*Anopheles arabiensis* and *Anopheles funestus*), as well as spatial correlations of the two variables within and between villages in rural Tanzania [25]. That study also showed that high indoor vector densities cluster in locations where houses with highest occupancy are also clustered, suggesting the possibility of relying on regular census data to predict malaria transmission patterns. Similarly, Smith et al. [26] illustrated that even though biting risk from malaria vectors may be highest at the edge of the villages near aquatic habitats, infectious proportions of these mosquito populations (tending to be the older ones) are far more likely to be clustered in the middle of the villages, with peak populations of the infectious ones occurring when overall vector densities start declining [26].

Despite the above observations, and other evidence suggesting that house design, geographical location of the house within settlements, and characteristics of surrounding environments all influence malaria transmission [16–19], several questions remain unanswered, particularly with regard to community awareness of the same factors. For example, it is unclear whether community members fully understand the importance of such factors, whether they have any concerns about them, and what their counter-efforts are during site selection and house construction. Yet, as malaria transmission declines, control efforts aiming at elimination should target locations and possibly households with greatest risk [27–29], while also actively engaging members of those communities and households.

The aims of this study were therefore: (a) to identify common house characteristics that may be associated with increased vector biting risk, and (b) to assess community knowledge and experiences regarding housing and environmental factors that influence malaria transmission in four rural Tanzanian villages and how these experiences can be incorporated in ongoing and future intervention options.

## Methods

### Study area

**Fig. 1:**
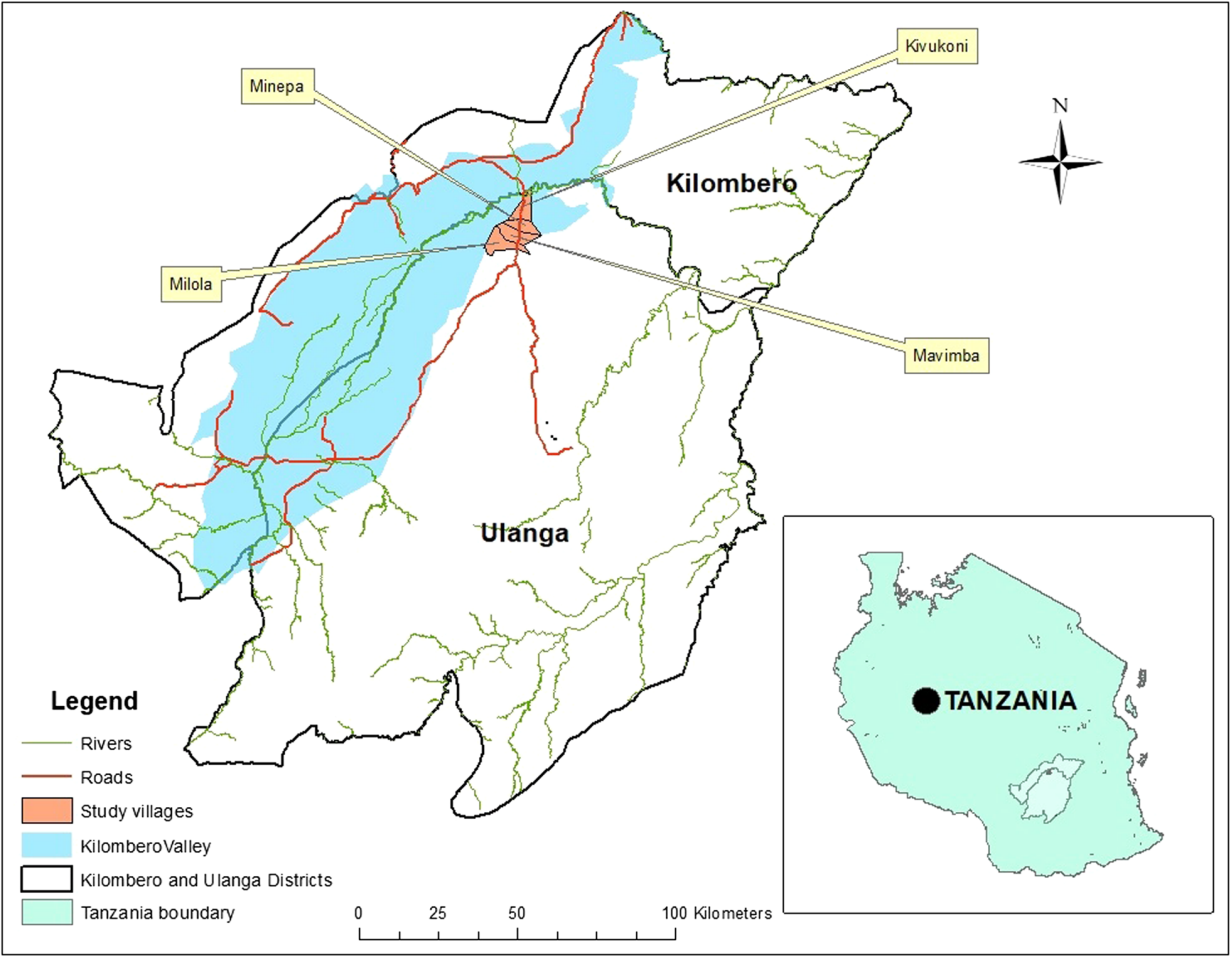
Map of the study villages. The study was conducted in households across four villages in Ulanga district, south of the Kilombero river in south-eastern Tanzania

**Fig. 2:**
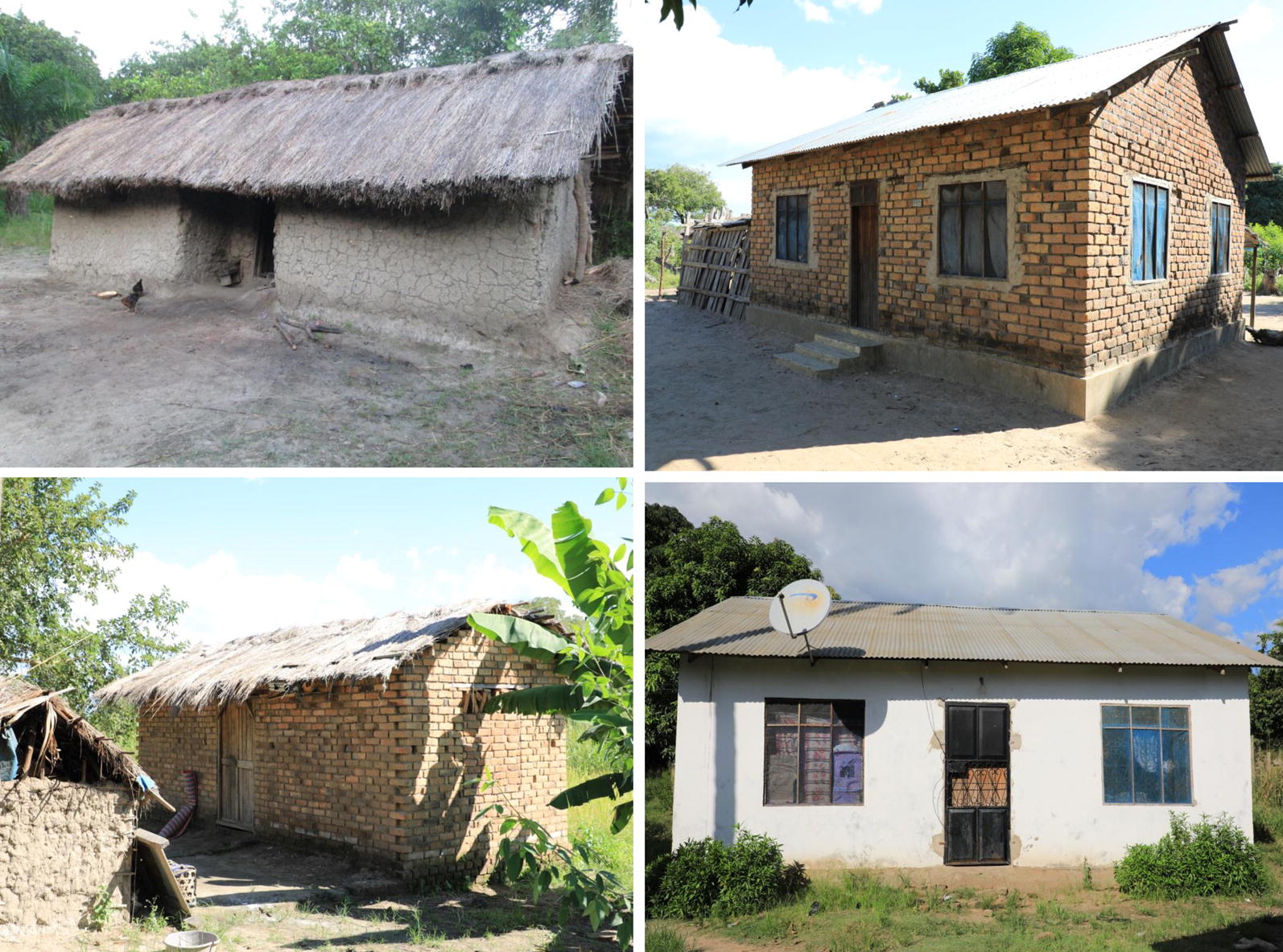
Pictorial representation of typical local house types in the study area. Top left: a house with grass thatch roofing with mud walls. Top right: a house with corrugated iron roof and brick walls not plastered on the outside, and sometime also not plastered on the inside. Bottom left: grass thatch roof with brick walls not plastered on the outside, and sometime also not plastered on the inside. Bottom right: a house with iron sheet roofing and plastered brick walls. A variety of window and door designs and covers are also illustrated

The study was conducted in four villages of Kivukoni (8.2135°S, 36.6879°E), Minepa (8.2710°S, 36.6771°E), Mavimba (8.3124°S, 36.6771°E) and Milola (8.2135°S, 36.6878°E) in Ulanga district, south-eastern Tanzania (Fig. 1). The local houses have either mud or bricks walls. The roofs are either grass-thatched or covered with corrugated iron-sheets, and most houses have open eaves (Fig. 2). Mean household size is 4.2 [30]. This area is perennially meso-endemic for malaria, and has high mosquito densities throughout the year, peaking between March and May. Annual rainfall and mean daily temperatures range from 1200 to 1800 mm and 20 to 32.6 °C, respectively.

The primary malaria vectors are *An. funestus* and *An. arabiensis,* with minor contributions from *Anopheles rivulorum* [31]. Malaria transmission intensities, i.e. entomological inoculation rate (EIR) was last estimated at 4.2 and 11.7 infectious bites/person/year (ib/p/yr) by *An. arabiensis* and *An. funestus,* respectively, totalling 15.9 ib/p/year [31]. The main vector control method is LLINs, usually distributed through mass campaigns done every 3–4 years, and keep-up campaigns done through reproductive health clinics. The main malaria vectors are resistant to pyrethroids used in the LLINs, but still susceptible to organophosphates [31].

### Study procedures

**Fig. 3:**
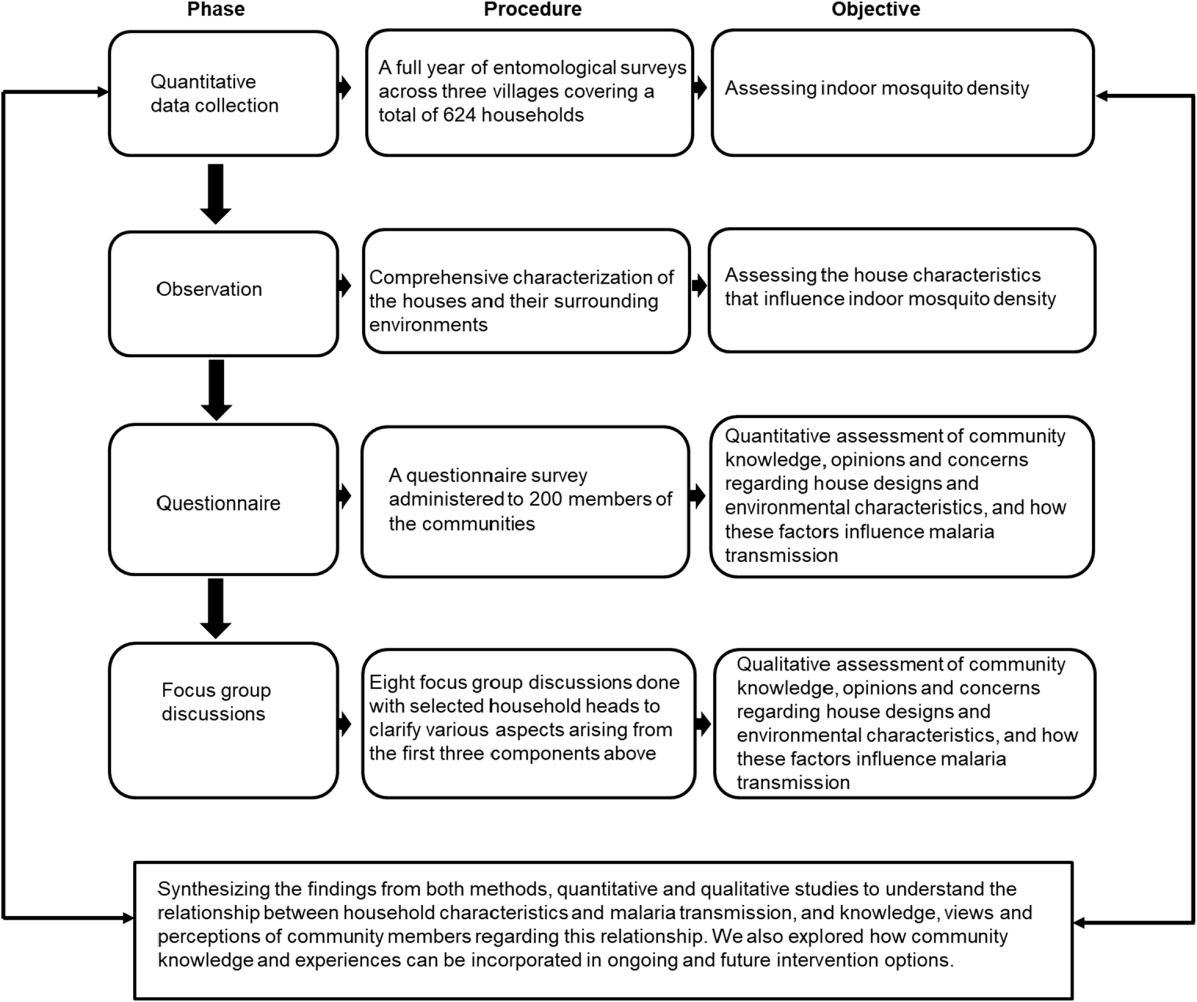
Mixed methods study design

The study used a mixed methods approach with four components (Fig. 3) including: (i) longitudinal surveys of indoor mosquito densities in sentinel and randomly selected households within the Ifakara Health and Demographic Surveillance System (HDSS) area [30] from January 2015 to January 2016; (ii) characterization of the houses and surrounding environmental variables; (iii) a questionnaire survey administered to community members to assess their concerns about household and environmental factors and whether they consider such variables when they are constructing their houses; and (iv) focus group discussions to assess people’s knowledge and perceptions on relationships between house characteristics and environmental factors associated with transmission.

#### Longitudinal surveys of indoor mosquito densities

Random sampling with replacement was done using household listings originally obtained from the Ifakara Health & Demographic Surveillance Systems unit (2433 houses) covering the study area. An initial set of 48 houses were selected and recruited upon consent of household heads, and were set as fixed sentinel houses where vector surveillance was conducted once every month for 12 months. Each month, another set of 48 houses was selected randomly from the same villages (with approximately similar spatial distribution as sentinel houses) for a one-off mosquito collection that month. The following month a new set of 48 houses were selected without repeating those that had previously been sampled. In the end, a total 624 households were studied over the 12-month period, with the 48 sentinels sampled 12 times each.

Since the three study villages were different in size and number of households, the actual selection and distribution of the sampling houses was done in such a way as to cover the full geographical extents of the entire study area. Then stratified random sampling was used. First, 1600 households were randomly identified and spatially assigned to 16 geographical clusters, each having 100 households. The sampling clusters were defined based on household latitudes so that clusters 1–16 were on a North–South direction. On the first month of study, the geographical clusters were visited and six households selected randomly per cluster. The six household heads were requested to volunteer in the study, and half of them requested for permission so their houses could be enrolled as fixed sentinel sampling stations for repeated monthly mosquito collections. Whenever a household head did not consent, the next household in the random listing was selected, so that there were always six households per cluster (three of them being fixed sentinels and another three being random households for one-off sampling). This way, there were 48 fixed sentinels throughout the study period and another 48 new houses newly selected each month across the clusters.

Thereafter, mosquito collections were conducted monthly in each of the study households, each time selecting a new set of three random households from each cluster. A weekly schedule was followed, working for 4 weeks/month. Each week, mosquitoes were sampled in four of the 16 geographical clusters, by visiting all six households per cluster each night, working for four nights per week. This way, all the 16 clusters were covered once every month. In each household, one occupied room was selected for assessing indoor mosquito densities. CDC light traps set near occupied bed nets were used [32], and were operated from 18:00 h to 06:00 h. Backpack aspirators (CDC model 1412-12 VDC 17 AmpHr Gel-Cell battery) were also used to sample resting mosquitoes each subsequent morning. The collected mosquitoes were killed in a closed container using petroleum fumes, then morphologically identified using taxonomic keys for *Anopheles* Africa, south of Sahara, developed by Gillies and Coetzee [33]. All samples were then sorted by sex, taxa and physiological status. Sub-samples of *Anopheles gambiae* complex were further identified by multiplex polymerase chain reaction (PCR), to identify sibling species using protocols developed by Scott et al. [34]. Also a sub samples of *An. funestus* were identified by using PCR, following the techniques developed by Cohuet et al. [35], and Koekemoer et al. [36].

#### Characterization of the houses and their environments

At each initial visit, all the 624 human houses were characterized from where mosquito collections had been done over the 1-year period. Geo-positions (latitudes and longitudes) of all the households were captured using hand-held GPS receivers (Magellan eXplorist 110) and recorded. In each house, the condition of windows, doors and walls were examined, and whether the houses had closed or open eaves. The surroundings of the households were examined and recorded any nearby water bodies, cattle sheds or other animal sheds. Other environmental variables recorded included whether the houses were located at the edge or in the middle of the villages and whether people kept animals, such as cattle, pigs or chicken.

#### Survey questionnaires: quantitative assessment of community knowledge, opinions and concerns regarding house designs and environmental characteristics, and how these factors influence malaria transmission

A structured questionnaire was developed, pretested and used for this survey. The questionnaire had three components (Table 1): the first component captured socio-demographic characteristics of participants including variables such as age, gender, education, marital status, household size, household income and expenditure. The second component examined the general knowledge of the participants on malaria, malaria transmission, its symptoms, preventive measures and care-seeking behaviours. The third component of the questionnaire assessed participants’ knowledge on how various house characterizes and environmental variables that might influence malaria transmission, how the participants currently deal with those associations and whether the participants usually put in place any measures to address specific malaria risk factors associated with housing and the environment.

**Table 1:** Description of the main themes addressed in the survey to assess community knowledge, opinions and concerns regarding house designs and environmental characteristics, and how these factors influenced malaria transmission

	Concepts investigated	Specific questions asked by the interviewer	Relevance of the concepts
1	Knowledge and perception about house characteristics, mosquito entry and malaria transmission risks	Do you know if the house design can influence mosquito entry?	Assessment of knowledge and perception of malaria transmission risks in relation to house characteristics
		Does your house allow mosquito entry?	
		If your house does not allow mosquito entry, how do you prevent mosquito entry?	
		Why does your house allow mosquito entry?	
		How do mosquitoes enter your house?	
		When was your house constructed?	
		Why did you decide to construct this kind of house?	
		What did you consider during construction?	
2	Knowledge and perception about environmental variables influencing mosquito density	Do you know if the environments surrounding your house influence mosquito density?	Assessment of knowledge and perception of malaria transmission risks in relation to environmental characteristics
		How does the environments surrounding your house influence mosquito density?	
		Mention the common mosquito breeding sites in your area	
		What do you do to prevent mosquito bites?	
3	Knowledge and perception about settlements, mosquito density and malaria transmission risks	Do you think the number of houses in an area can influence mosquitoes and malaria transmission?	Assessment of knowledge and perception of malaria transmission risks in relation to settlement patterns
		Do you think constructing houses near other houses or far from other houses is an important factor in regard to mosquito biting risk and malaria transmission?	
		Why do you think close house have many mosquitoes?	
		What can be done to control mosquitoes in such kind of environment?	

The questionnaires were translated and administered in Kiswahili, which is the common language spoken by communities across the study area. After pre-testing and correcting all issues arising, the questionnaire was administered by trained research officers to consenting household heads across three study villages where the entomological surveys had been done and in one village where there was no entomological surveillance. A total of 200 participants responded to the survey.

#### Focus group discussions: qualitative assessment of community knowledge, opinions and concerns regarding house designs and environmental characteristics, and how these factors influence malaria transmission

Study participants were selected from among the households participating in the entomological survey and recruited upon consent with help from the community leaders. A total of 12 people were recruited for each focus group, ensuring all participants were from the same village.

Eight focus group discussions (FGD) were conducted across the four study villages, each FGD comprised 12 participants recruited from the same village. Overall the study had a total of 96 participants aged between 18 and 70 years. There was an equal representation of men and women in each of the FGD groups (6 men and 6 women). The discussions were held between January and March 2017 and were conducted at local primary schools within the respective villages.

A study guide for the FGD was developed and pretested. The FGD guide followed the themes described in Table 1 for the questionnaire surveys. Adults and youths were separated during the interviews to avoid any chance of intimidation during the discussions. The purpose of the discussions was explained and verbal and written informed consents were obtained from the participants. The discussions were semi-structured: the facilitator asked a question and the participants discussed their knowledge, views and comments. The discussions were facilitated by two social scientists; a note taker and an interviewer, and were tape recorded to ensure that all information was captured. Discussions were all conducted in the local language (Swahili), and lasted between 40 and 60 min each.

The study assessed whether people think that the house designs matter in terms of the risk they are exposed to. The study then assessed if people care about environmental variables and if they worry about these factors when they construct their houses. In cases where people were concerned, the study also assessed how they cope with these concerns. Another concept investigated during the FGDs was whether participants were aware of any relationship between settlement patterns and malaria transmission. For example, the study examined whether the participants thought constructing houses near other houses or far from other houses was an important factor in regard to mosquito biting risk and malaria transmission.

### Data analysis

#### Analysis of quantitative data

Data were analysed using open source software, R version 3.1.0, using the *lme4* package [37]. Associations between house characteristics and indoor mosquito densities were examined by generalized linear mixed effects models (*glmer)* [38], by fitting the data in log-linked Poisson error distributions. The indoor densities of *An. arabiensis*, *An. funestus*, *Culex* and *Mansonia* species were modelled as a function of different house and environmental characteristics. This study assessed effects of (a) wall type, (b) roof type, (c) window type, (d) whether chickens were kept inside or outside the house, and (e) whether the eave spaces were closed or open. Date of mosquito collection and house identification numbers were incorporated as random variables in the *glmer* models. Estimated mean indoor mosquito densities per house per night, the relative rates (RR) of observing these mosquito catches, and associated 95% confidence intervals (CI), were computed from exponentials of coefficients generated from the *glmer* models.

The monthly temporal patterns of biting risk by the two malaria vector species and also the culicine genera, i.e. *Culex* spp. and *Mansonia* spp. mosquitoes were presented graphically.

Data generated through the questionnaire surveys were summarized and presented descriptively in tables to capture: (a) knowledge about risk factors, (b) whether people worry about house and environmental characteristics and (c) whether they consider such variables when constructing their houses.

#### Processing and analysis of qualitative data

After the FGD session, all recordings were retrieved from the tape recorder and archived on a password-protected computer. A trained behavioural scientist then transcribed and translated the data from Kiswahili to English for further analysis. The scripts were then imported into NVivo software version 13 for organizing and indexing the data. Emergent themes were organized to assess: (a) whether people worry about the characteristics of the house and if they consider such variables when constructing their houses, (b) whether people care about environmental variables and if they consider such variables when constructing their houses, (c) whether people know effects of seasonality on malaria transmission, (d) whether people know the relationship of settlement patterns and human population on malaria transmission, and (e) how the people cope with these risk factors if they are actually aware of relationships with malaria transmission. Direct quotes from the participants are presented to support their responses on the themes.

## Results

### Indoor densities of malaria and non-malaria mosquito vectors

**Fig. 4:**
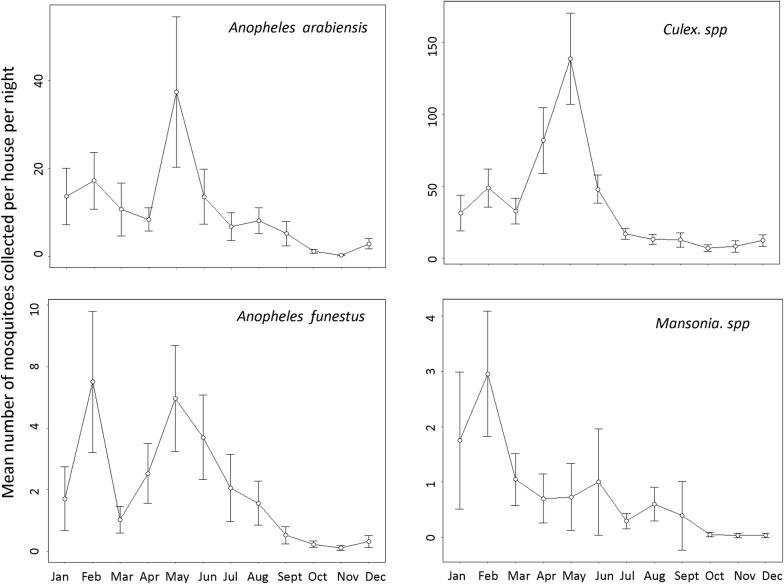
Monthly trends of mean number of mosquitoes of different species collected per house per night. The Y-error bars represent 95% CI. All the species generally followed same trend peaking between April and June, except *Mansonia* spp., whose densities peaked between January and March

A total of 129,052 mosquitoes were collected by CDC light traps and backpack aspirators indoors. Of these, 19.9% were *Anopheles* (n = 25,670) and 80.1% culicines (103,382). The 25,670 *Anopheles* mosquitoes included 20,254 *An. arabiensis* (78.9%) and 4800 *An. funestus* (18.7%), 205 *Anopheles coustani* (0.8%), 128 *Anopheles pharoensis* (0.5%), 103 *Anopheles squamosus* (0.4%), 77 *Anopheles ziemanni* (0.3%) and 103 *Anopheles wellcomei* mosquitoes (0.4%). Of 103,382 culicine mosquitoes collected, there were 100,047 *Culex* spp. mosquitoes (96.7%), 319 *Mansonia* mosquitoes (3.1%) and 138 *Aedes* mosquitoes (0.1%). The highest densities of host seeking mosquitoes were observed in Minepa village, where about 54% and 65% of all *An. arabiensis* and *An. funestus* mosquitoes were collected. The mean density (± se) of *An. gambiae* caught per village per night were, as follows: Minepa, 65 ± 20, Kivukoni, 19 ± 6 and Mavimba, 8 ± 7. Mean monthly densities of the indoor mosquito catches are shown in Fig. 4. Densities of *An. arabiensis* and *An. funestus* peaked between April and June in all study villages but were generally low between September and November (Fig. 4).

### Physical characteristics and microclimate of sampled houses

A variety of construction materials were observed in the local houses (Table 2). Most houses had walls made of mud (50.5%), un-plastered brick (48.6%) or brick and concrete plastering (1.0%). With regard to roofs, a significant proportion had grass-thatched roofing (60.6%), while the rest had corrugated iron sheet roofing (39.4%). At the time when mosquito traps were being set in the evenings, we observed that 64% of the houses had open doors at that time, possibly letting in mosquitoes.

**Table 2:** Physical characteristics and microclimatic conditions in sampled houses

Variables assessed	Category	Percentage (N)
Wall type	Plastered brick walls	1.0% (6)
	Mud walls	50.5% (315)
	Un-plastered brick walls	48.6% (303)
Eave space	Closed	25.3% (158)
	Open	74.7% (466)
Roof type	Corrugated iron sheet	39.4% (246)
	Grass-thatched	60.6% (378)
Window covers	With netting screen	25.3% (158)
	Without netting screen	74.7% (466)
Door (observed from 6 pm to 7 pm)	Open	63.9% (399)
	Tightly closed	29.5% (184)
	Partially closed	6.6% (41)

### Household risk factors associated with indoor mosquito densities

Houses with mud walls were significantly associated with higher vector densities compared to brick walls. *Anopheles arabiensis* densities were higher in mud-walled houses [RR = 3.9 (2.3–5.6), P < 0.005] and the number of *An. funestus* was also higher in houses with mud walls [RR = 4.0 (2.5–5.3.) P < 0.005]. *Culex* [RR = 2.3 (2.1–2.6), P < 0.001] and *Mansonia* species [RR = 2.5 (1.7–3.8), P < 0.005], were also more abundant in houses with mud walls than brick walls. Similarly, open eaves were significantly associated with higher indoor vector densities. There were more *An. arabiensis* [RR = 1.9 (1.8–2.0), P < 0.001] and more *An. funestus* [RR = 1.2 (0.9–1.6) P < 0.005] in houses with open eaves than houses with closed eaves. This study observed similar differences for *Culex* [RR = 1.9 (0.8–1.0), P < 0.001] and *Mansonia* species [RR = 1.5 (1.4–1.8), P < 0.001] in houses with open eaves relative to closed eaves. Tables 3 and 4 provide parameter estimates for different house characteristics and their significance in relation to mean catches of mosquitoes in the selected households. The number of *An. funestus* was significantly higher in houses with chicken indoors [RR = 2.0 (1.8–2.1), P < 0.001] compared to those without chicken, but no such association was observed with *An. arabiensis* [RR = 1.8 (1.8–1.9), 0.343]. Similarly, there were higher densities of *Culex* [RR = 1.2 (1.1–1.2), P < 0.001] and *Mansonia* mosquitoes [RR = 2.6 (2.4–2.7), P < 0.001] in houses with chicken indoors.

**Table 3:** Summary statistics for mosquitoes caught in houses with different characteristics. The mean nightly catches of *An. arabiensis* and *An. funestus* mosquitoes, relative rates and the associated significance levels were calculated from the generalized linear mixed models (GLMMs) at 95% confidence interval

Variables	Category	*Anopheles arabiensis*			*Anopheles funestus*		
		Mean (CI)	RR (95% CI)	P value	Mean	RR (95% CI)	P value
Wall type	Bricks	9.3 (6.9–25.5)	1.0		3.8 (2.4–5.2)	1.0	
	Mud	17.7 (10.1–22.3)	3.9 (2.7–5.6)	0.005	5.0 (2.6–7.4)	4.0 (2.4–5.3)	0.011
Eave space	Closed	9.3 (4.2–14.4)	1.0		2.8 (1.3–4.2)	1.0	
	Open	19.2 (12.9–25.6)	1.9 (1.8–2.0)	< 0.001	4.9 (3.2–6.7)	1.2 (0.9–1.6)	0.005
Roof type	Iron sheets	14.4 (6.6–22.2)	1.0		3.3 (1.9–4.8)	1.0	
	Grass	18.2 (11.8–24.6)	1.5 (1.4–1.7)	< 0.001	5.1 (3.0–7.1)	2.4 (2.0–3.0)	0.318
Doors	Closed	12.6 (0.0–16.8)	1.0		1.6 (0.0–3.0)	1.0	
	Open	36.0 (9.5–42.5)	1.4 (0.9–2.1)	< 0.001	3.8 (2.4–5.2)	1.6 (1.3–2.0)	< 0.001
Window	Screened	15.8 (5.4–23.3)	1.0		4.2 (2.7–5.7)	1.0	
	Unscreened	17.1 (11.5–26.6)	1.9 (1.7–1.9)	< 0.001	4.9 (1.7–8.0)	2.9 (2.3–2.7)	0.130
Chicken indoors	No	15.3 (9.5–21.0)	1.0		4.2 (2.6–5.9)	1.0	
	Yes	20.4 (10.7–29.9)	1.8 (1.8–1.9)	0.343	4.8 (2.4–7.1)	2.0 (1.8 –2.1)	< 0.001

**Table 4:** Summary statistics for the number of mosquitoes caught in houses with different characteristics

Variables	Category	*Culex species*			*Mansonia species*		
		Mean (CI)	RR (95% CI)	P value	Mean	RR (95% CI)	P value
Wall type	Bricks	51.7 (45.2 – 86.9)	1.0		0.6 (0.3 – 1.5)	1.0	0.805
	Mud	66.2 (43.9 – 88.5)	2.3 (2.1– 2.6)	<0.001	1.5 (0.4 – 2.5)	2.5 (1.7 – 3.8)	<0.005
Eave space	Closed	57.9 (27.7 – 88.1)	1.0		0.3 (0.1 – 0.6)	1.0	
	Open	67.2 (48.7 – 85.8)	1.9 (1.8 – 2.0)	<0.001	1.5 (0.7 – 2.3)	1.5 (1.4 –1.8)	<0.001
Roof type	Iron sheet	56.7 (33.9 – 79.4)	1.0		0.7 (0.1 – 1.4)	1.0	
	Grass	70.2 (48.7 – 91.7)	2.6 (2.4 – 2.7)	0.139	1.5 (0.6 – 2.3)	2.5 (1.8 – 2.4)	0.705
Doors	Closed	29.5 (0.0 – 79.9)	1.0		0.2 (0.0 – 0.9)	1.0	
	Open	56.2 (43.9 – 88.5)	2.3 (2.1– 2.6)	<0.001	1.5 (0.4 – 2.5)	2.5 (1.7 – 3.8)	<0.005
Window	Screened	55.4 (23.2 – 86.6)	1.0		0.9 (0.0 – 2.0)	1.0	
	Unscreened	68.4 (50.3 – 86.5)	2.5 (2.4 – 2.6)	<0.001	1.3 (0.6 – 2.0)	1.8 (1.7 – 1.9)	<0.001
Chicken indoors	No	63.8 (46.2 – 81.5)	1.0		1.1 (0.5 –1.8)	1.0	
	Yes	67.4 (34.6 – 100.0)	1.2 (1.1– 1.2)	<0.001	1.3 (0.0 – 2.7)	2.6 (2.4 – 2.7)	<0.001

The mean nightly catches of *Culex* and *Mansonia* species, relative rates and the associated significance levels were calculated from the generalized linear mixed models (GLMMs) at 95% confidence interval

### Socio-demographic characteristics of survey participants

In the questionnaire survey, the study participants were 40.5% males and 59.5% females, with overall age range between 18 and 95 years (mean age = 36.9 years). The majority had acquired primary education and were farmers. All the demographic characteristics are shown in Table 5.

**Table 5:** Socio-demographic characteristics of participants who responded to the survey questionnaire to assess people’s awareness of how house characteristics affect malaria transmission

Variables assessed	Category	Percentage (N)
Gender	Males	40.5 (81)
	Females	59.5 (119)
Age	18–35	48.7 (97)
	36–50	26.5 (53)
	51–65	15 (30)
	> 65	20 (10)
Marital status	Married	66 (132)
	Unmarried	20 (40)
	Widow/widower	4.5 (9)
	Divorced	9.5 (19)
Level of education	No formal education	11.5 (23)
	Primary school	67.5 (135)
	Secondary school	18 (36)
	College/university	2 (4)
	Other trainings	1 (2)
Occupation	Peasant (self-employed in agriculture)	68.5 (137)
	Small scale business	4 (8)
	Formal employment	0.5 (1)
	Unemployed	0.5 (1)
	Other	26.5 (53)

### Results of the questionnaire survey: participants’ knowledge regarding indoor mosquito density and how this relates to housing characteristics and environmental variables

Generally, most respondents associated malaria infections with poor house design features and physical conditions (Table 6). More than 90% of participants were aware of different environmental characteristics influencing mosquito density in their area. Similarly, 98.5% of respondents reported that their houses allowed mosquito entry. Houses with open eave space, unscreened windows and mud walls were generally associated with higher mosquito densities in the houses. The age of houses observed was fairly even, with 37% of the houses less than 4 years old, 37% more than 9 years old and the remainder being between 5 and 8 years old.

**Table 6:** Summary of community members’ knowledge about indoor mosquito density and how these relate to certain housing and environmental characteristics

Variables assessed	Response category	Percentage (N)
Whether participants believe their house let in mosquitoes	Yes	98.5% (197)
	No	1.5% (3)
How participants believed mosquitoes entered their houses	Through open doors	28.5% (57)
	Through windows	32.5% (65)
	Through holes in the wall	3.0% (6)
	Through the eave space	19.5% (39)
	Through both open windows and doors	13.5% (27)
	Through open window doors, holes in walls and eaves	2% (4)
	Others	1% (2)
Age of house (date of construction)	< 4 years ago	36.5% (73)
	5–8 years ago	26.5% (53)
	> 9 years ago	37.0% (74)
Main reasons for constructing the kind of house	Because it is permanent	36.5% (73)
	Because it prevents animals from entering the house	26.5% (53)
	Because it prevents insects	17.0% (34)
	Others	20.0% (40)
Whether people consider mosquito prevention as a key factor when constructing houses	Yes	58.5% (117)
	No	40.0% (80)
	Does not know	1.5% (3)
Specific practices considered by participants (during construction of their houses) for preventing mosquitoes	Netting on the window	38.0% (76)
	Blocking the eaves	8.0% (16)
	Using bricks on the wall	2.0% (4)
	Using cement on the wall	1 (2)
	Does not know	38.5% (77)
	Others	12.5% (25)
Whether participants knew open eave spaces let in mosquitoes	Yes	96.0% (192)
	No	4.0% (8)
Whether participants knew that surrounding environments influence vector densities in their houses	Yes	97.0% (194)
	No	3.0% (6)

### Focus group discussions: participants’ knowledge regarding indoor mosquito density and how this relates to housing characteristics and environmental variables

In the FGDs, participants stated that house structure had an effect on number of mosquitoes entering their houses, and consequently on malaria transmission. The participants were concerned about living in poorly constructed, small houses with poor lighting and gaps on the eaves, walls, windows and doors. They mentioned that these gaps allow mosquito entry into their houses and expressed concern that they are frequently bitten by mosquitoes while indoors when they are not under bed nets. Most participants associated this situation with low household income, expressing concerns that they did not have enough money to construct proper houses.

*“Many mosquitoes are found in those houses with smaller windows, in which there is less light because mosquitoes like dark places. Then mosquitoes enter the house and won’t leave the area”. (46* *years old male, Milola village).*

Other house characteristics implicated in mosquito biting risk were nearness to mosquito aquatic habitats and also height of the house. The participants suggested that it was necessary to construct houses with greater heights and far from water bodies to prevent this menace. Here are two examples of quotes from one of the participants on this subject.

“*It is important to make sure that houses are built as far away from the mosquito aquatic habitats as possible. It is good to know the distance that mosquitoes can fly from their aquatic habitats so that we can know where to build houses”.* (37 years old male, Minepa village).

“*It is necessary to build a house that makes it hard for mosquitoes to get inside. I know that houses that are built lower let mosquitoes in than the houses that have larger height”.* (26 years old male Milola village).

There was also a strong linkage of environmental conditions with mosquito densities in houses. It was commonly mentioned that the proximity of the mosquito aquatic habitat and the state of the environment surrounding the houses had a lot of influence on the mosquito density, and consequently with higher number of malaria cases. Participants stated that mosquitoes liked to rest in dark, damp, grassy and bushy areas. Others said that mosquitoes liked to accumulate in dirty environments as the dirt and trash provided mosquitoes with good hiding places. There were general references to “dirty water”, by which participants mostly meant stagnant water pools outdoors (“dirty water” = water not fit for drinking). This type of water, found near dwellings was commonly mentioned as being related to mosquito aquatic habitat. There was however no evidence that the community members knew the specific characteristics of aquatic habitat used by malaria mosquitoes as opposed to other mosquitoes. For example, two of the participants said:

“*If the environment is dirty mosquitoes increase in number, because they are able to rest in trash. That means if we clean our surroundings then there will be very few mosquitoes. But if our environment is dirty mosquitoes reproduce and increase in numbers”* (58 years old male, Minepa village).

“*Mosquitoes are found in dirty water around the house, like in water that is in broken containers or cups. They are also found in the ponds, shrubs and in dark places. I believe that is where they accumulate”* (34 years old female, Mavimba village).

An interesting theme that arose during the FGDs was how behaviours of neighbours was thought to influence vector densities and malaria transmission in houses that are in close proximity. Some participants thought that if the behaviours and practices of their neighbours were encouraging mosquito accumulate and survival, then those mosquitoes would also go to the other surrounding houses as well.

“*When there are many houses together, it means that there is a lot of trash and other things in which mosquitoes can rest. For example, I may keep my surroundings clean, but if my neighbours do not clean their surroundings, and they live close to me, then mosquitoes can easily come from their houses to mine, it will make it easy for mosquitoes to bite me”.* (37 years old female, Milola village).

Participants perceived that nucleated settlements encouraged malaria transmission more than dispersed settlements. Most participants expressed that when households were close to each other, mosquitoes could easily move from one house to another, easily transmitting malaria. According to the participants, when households are far apart then it would take time for mosquitoes to travel between the households. Here are comments from some of the participants:

“*If there are many houses close together then mosquitoes can move from one house to another. But if houses are few and far apart then the mosquitoes cannot really get from one house to another easily, because there can be many obstacles for them on the way, like the wind and rain”* (40 years old female, Milola village).

“*When there are a lot of houses together then it is a problem for us because this way many mosquitoes come, because they know that they can get a lot of people in close proximity. It is easy for the mosquitoes to transmit malaria” (23* *years old male, Kivukoni village).*

“*If houses are scattered, then the wind will blow mosquitoes to other places where they cannot easily get people”* (51 years old male, Milola village).

Insecticide-treated bed nets were the main form of protection used against mosquito bites and malaria transmission. All participants reported that they used bed nets regardless of the season. Other means of protection mentioned were cleaning the environment, drying up ponds when possible, covering up with clothing when people are outdoors, using insecticide repellent lotions and indoor sprays and going indoors early. There were however concerns about the integrity of the available nets and how this influences their efficacy. Examples of relevant quotes included:

“*One thing that most of the people in the village can afford is to use bed nets, because everyone has bed nets here. But in order for these nets to be useful they have to be in a good shape. Sadly not everyone has new nets. I sleep with my children in the same bed because we have one net, but our net is not in a very good state, it has a lot of holes that sometime let mosquitoes in. But it still helps; it is not the same as if we did not use anything”* (31 years old female, Kivukoni village).

*“We make sure that children are protected from mosquito bites by making sure they go to bed early when it gets dark, and then we make sure they are covered by bed nets. Sometimes when they are sitting outside then we use a piece of cloth to chase mosquitoes away*” (54 years old male, Milola village).

Lastly, there was generally a high level of understanding regarding seasonality and malaria transmission. Participants mentioned that though mosquito numbers were generally high in the area, most biting and malaria transmission occurred in the rainy season, especially February through May, during the long rains. Here are some of the responses from the participants:

“*Mosquitoes are found here throughout the year. However, their population is the highest during the rainy season because there is a lot of water around, hence many* aquatic habitat*. During the dry season there are fewer places where mosquitoes accumulate, so their population is lower”* (28 years old male, Milola village).

“*There are a lot of mosquitoes from February to April because this is the time where there is a lot of water everywhere. There are many pools, ponds and bushes where mosquitoes can accumulate. The mosquito population starts to go down from May and June when the weather starts to get dry and cold”* (24 years old male, Minepa village).

## Discussion

Housing design has been shown in different studies to significantly influence malaria transmission [30–32]. Most people in rural and peri-urban areas live in poorly finished houses with open eaves, doors and windows, hence exposure to malaria transmission is very high. Mosquitoes enter the houses easily through eaves and other openings, thus increasing likelihood of biting and pathogen transmission [29, 30]. This study found that the number of mosquitoes were significantly higher in houses with open eaves, grass roofs, Mud walls and unscreened windows. Furthermore, keeping chickens inside the house was also associated with high number of mosquitoes. Unfortunately, these communities cannot afford essential house construction and mosquito-proofing materials. Greater sensitization and enabling financing initiatives to promote better housing should therefore be considered in vector control initiatives to accelerate malaria elimination efforts. This study found statistically higher indoor densities of the main malaria vectors (*An. arabiensis* and *An. funestus*) in houses with mud-walls as opposed to plastered or brick walls, open as opposed to closed eaves, and also in unscreened as opposed to screened windows. Keeping chickens inside the house was similarly associated with higher indoor densities.

There was already very high levels of awareness about how housing characteristics and the surrounding environment influence mosquito densities, and consequently malaria transmission. Another factor identified was the small size of houses (area and height). In additional to poorly-constructed houses with gaps in the eaves, walls, windows and doors. However, people were constrained by low income and could not readily afford construction of properly ventilated and mosquito-proof houses. Nearly all respondents reported that their houses allowed mosquito entry, but they could not afford modern building materials. They expressed that they are also being bitten by mosquitoes while indoors when they are not under bed nets. The relationship between poor housing and low-income is expected in such settings. However, although many households in rural areas are unable to afford modern housing, there are cheaper and simple ways of modifying or screening houses which could readily prevent mosquito entry, especially if the community members are adequately sensitized and financial barriers removed, e.g. through subsidies. Unfortunately, malaria control authorities in sub-Saharan Africa have not invested in house improvement programmes despite the significant available evidence [39, 40].

Community knowledge on mosquito aquatic habitat is another important factor to consider for successful community-based vector control. In this study, participants of the FGD were identified various sources of mosquitoes in their surroundings, such as stagnant water pools, dense vegetation and rice fields. Studies elsewhere have yielded similar findings [41, 42], and such high levels of community awareness can be harnessed to improve disease prevention and control initiatives. In rural south-eastern Tanzania, the nucleated settlement patterns, combined with the previously described spatial distribution of vector densities across the villages, indicate that targeted interventions if supported by local community champions could indeed be highly effective. A study done by Mwangungulu et al. in the same villages yielded evidence that community members are capable of identifying places with low, medium and high mosquito densities [22]. Mosquitoes accumulate in shallow pools of water and puddles [43, 44], which are numerous in rural areas especially during the rainy season. Draining pools of water, levelling land, construction of drains and proper waste management could eliminate mosquito aquatic habitat around homes [45, 46], and subsequently reduce malaria transmission [47]. Given the existing high-levels of awareness, further control initiatives involving environmental management could be improved if community members are considered the main stake holders. Such community-based approaches could also be used to scale-up better housing designs and access to commodities necessary for screening windows, eaves and doors even in rural, low-income areas.

Community knowledge, perception and practices are very important for designing or improving disease control programmes and identifying factors for effectiveness of the interventions [48]. Promoting community based programmes can help to achieve population level change in risk behaviours which in turn results into positive changes [49]. Unless communities recognize the importance of changing behaviours which influence disease transmission, the best designed interventions might not achieve the intended goals. More broadly in primary health care, it has been demonstrated in places such as Tigray, Ethiopia, that teaching mothers about home-based malaria treatment and management can vastly improve treatment outcomes and reduces child mortality [50]. In this particular programme in Ethiopia, reduction of up to 40% in under-five mortality was achieved in holoendemic malaria areas, by simply training and relying upon local mothers to teach each other how to dispense anti-malarial medicines to their children.

Another important point that emerged from this study was participants’ impression that if the houses are close to each other, malaria transmission is likely to be higher because mosquitoes can easily move between houses, and spread pathogens. Though participants were not able to describe settlement patterns, they perceived that closer and congested household are at higher risk of malaria transmission, and were concerned with the distances that a mosquito can move from one house to another. Previous study suggested strong correlations between household occupancy and malaria vector densities [25], but no specific analyses have been done on effects of distance between households on malaria transmission. By combining the new findings from this current study with the observations previously described by Mwangungulu et al. [22] and Kaindoa et al. [25], community driven intervention could be designed, which relies on trained locals to support environmental management and house improvement initiatives for mosquito control [51]. For example, community-based artisans, if provided minimal government support, could create local businesses that support sustainable house-improvement programs with potential benefits against vector-borne diseases.

## Conclusion

To improve malaria control and elimination efforts, it is important to develop an understanding of community perspectives on persistent malaria transmission within and around their villages, then engage these communities in identifying and addressing the key factors contributing to the persistent transmission. This study has shown that in rural south-eastern Tanzania, significant proportions of people still live in houses with open eaves, unscreened windows and gaps on doors. Though the people are fully aware of associated mosquito biting and pathogen transmission risks, they are constrained by low-income levels and cannot readily afford better housing or house improvement (e.g. through screening of eaves and windows), alongside other competing priorities. This study concludes that community-based house improvement initiatives combined with targeted subsidies could lower the financial barriers, improve access to essential construction materials or designs, and significantly accelerate malaria transmission control in these communities.
