# Correction to: Housing gaps, mosquitoes and public viewpoints: a mixed methods assessment of relationships between house characteristics, malaria vector biting risk and community perspectives in rural Tanzania

**DOI:** 10.1186/s12936-018-2473-4

**Published:** 2018-09-10

**Authors:** Emmanuel W. Kaindoa, Marceline Finda, Jepchirchir Kiplagat, Gustav Mkandawile, Anna Nyoni, Maureen Coetzee, Fredros O. Okumu

**Affiliations:** 1 Environmental Health and Ecological Science Department, Ifakara Health Institute, P. O. Box 53, Ifakara, Tanzania; 2 School of Public Health, Faculty of Health Sciences, University of the Witwatersrand, Johannesburg, South Africa; 3 Institute of Biodiversity, Animal Health and Comparative Medicine, University of Glasgow, Glasgow, G12 8QQ UK; 4 School of Medicine, College of Health Sciences, Moi University, Eldoret, Kenya; 5 Wits Research Institute for Malaria and Wits/MRC Collaborating Centre for Multidisciplinary Research on Malaria, School of Pathology, Faculty of Health Sciences, University of the Witwatersrand, Johannesburg, South Africa; 6 Centre for Emerging Zoonotic & Parasitic Diseases, National Institute for Communicable Diseases, Johannesburg, South Africa

## Correction to: Malar J (2018) 17:298 10.1186/s12936-018-2450-y

Following publication of the original article [1], the author flagged that the clause “and competing household priorities” was missing from the second sentence of the conclusion section of the Abstract; while this clause was in the Conclusion section of the main article text.

The consequence of this error was that there was a discrepancy between the implications of the Abstract conclusion and that of the main Conclusion of the article.

As such, the article [1] has now been updated to modify this error such that “and competing household priorities” is in the Abstract conclusion as well as the Conclusion of the main article text.

We apologize for this processing error.
